# Early Life Predictors of Socio-Emotional Development in a Sample of Egyptian Infants

**DOI:** 10.1371/journal.pone.0158086

**Published:** 2016-07-05

**Authors:** Ammal M. Metwally, Ebtissam M. Salah El- Din, Manal A. Shehata, Ashraf Shaalan, Lobna A. El Etreby, Wafaa A. Kandeel, Sanaa Y. Shaaban, Thanaa M. Rabah

**Affiliations:** 1 Department of Community Medicine Research, Medical Division, National Research Centre, Giza, Egypt; 2 Child Health Department, Medical Division, National Research Centre, Giza, Egypt; 3 Biological Anthropology Department, Medical Division, National Research Centre, Giza, Egypt; 4 Pediatric Department, Faculty of Medicine, Ain Shams University, Cairo, Egypt; TNO, NETHERLANDS

## Abstract

**Introduction:**

Emotional problems are amongst the most critical concerns to be intentionally handled to enhance the wellbeing and development of children.

**Objective:**

To determine the predictors of socio-emotional development of Egyptian infants related to infant feeding practices, aspects of infant and maternal health and socioeconomic status.

**Subjects and Methods:**

A cross-sectional comparative study included 322 breast fed, 240 bottle fed and 93 mixed fed infants, from 6–24 months of age, who were enrolled in the Well–Baby Clinic of the National Research Centre and from pediatric outpatient facilities in urban Cairo. Assessment of socio-emotional development was performed using Bayley Scales of Infant and Toddler Development (Bayley III). Detailed maternal and infant history was recorded. Levels of serum zinc, copper, iron, vitamin B12 and complete blood count (CBC) were assessed in a subsample of 193 infants.

**Results:**

The risk of having below average socio-emotional composite score was nearly two and half times among formula-fed infants than among breast-fed infants. By binary logistical regression analysis, predictors of below average socio-emotional score were a lower serum zinc value, being formula fed during the first half-year and introduction of complementary food before the age of six months (p< 0.05).

**Conclusion:**

Exclusive breastfeeding and to a lesser extent mixed feeding during the first half year is correlated with above average socio-emotional development. Maternal education and zinc status were also determinants of better infant mental health. Our endeavors ought to be directed towards integrated interventions addressing multiple risks to children’s development.

## Introduction

Socio-emotional development during infancy and early childhood has been described as “the emerging capacity of the child to experience, control, and express feelings; form close and secure interpersonal connections; investigate the environment and learn, all in the setting of family, society and cultural anticipations” [1, 2].

As infants develop, emotional expressions become better-organized, partly due to caregivers’ supportive backing. Throughout the first two years, infants become progressively capable of understanding their own and others’, emotions, engaging in purposed communication and sharing deliberately with others [3, 4, 5].

Saarni et al. [6] Described four phases of the development of emotional communication between the infant and an adult. Phase 1 (prenatal to 6 weeks) describes the infant’s valenced reactions to emotion signals. Phase 2 (6 weeks to 9 months) focuses on the pre-referential communication, where the infant can engage in synchronous dyadic interaction with the caregiver. Phase 3 (9 months to 18 months) covers the development of referential emotion communication, behavioral regulation (i.e., where the expressive behaviors of the child are affected by the other’s emotional expressions). Phase 4 (18 months/2 years) is marked by the development of what is called self-conscious emotion (e.g., shame, guilt, pride).

Mental health disorders in infants and toddlers might be reflected as physical symptoms, overall delayed development, inconsolable crying, sleep problems, aggressive or impulsive behaviour and paralyzing fears [7]. Early childhood adversity or untreated mental health disorders can hinder children’s socio-emotional development aggravating early learning, social relationships, and deep-rooted wellbeing [8, 9]. Some early mental health disorders have lasting effects and may appear to be precursors of mental health problems in later life, including withdrawal, sleeplessness, or lack of appetite due to depression, anxiety, and traumatic stress reactions [10]. In Egypt, it was estimated that about 14% of the total burden of ailment were psychological well-being connected conditions [11].

Numerous variables may affect a child’s socio-emotional development [12]. Development relies on accessibility of sound nourishment [13], healthy environment [14], kind and thoughtful cooperation via parental figures, opportunities for learning and standard training and group support [15]. These elements have synergistic effects to promote proper child development [13].

### Nutrition

Adequate nutrition (adequate energy, protein, fatty acids, and micronutrients) is essential to fuelling early childhood's rapid brain growth. The well-nourished child is better able to interact with the caregivers and environment in a way that provides the experiences necessary for optimal brain development [16].

Undernutrition impairs physical growth, physical activity, and motor development, which may, in turn, influence brain development through two pathways. The first pathway is through caregiver behavior and the second is through child exploration of the environment. First, caregivers may not provide age-appropriate stimulation for small for age or malnourished children, which could result in altered brain development. Also, undernourished children may be frequently ill and therefore fussy, irritable, and withdrawn, leading caregivers to treat them more negatively [17]. Reduced activity due to undernutrition may limit the child's exploration of the environment and initiation of caregiver interactions, which could also lead to poor brain development. [18].

### Social Factors

Children’s families, schools, neighborhoods, peers and culture all play a role in emotional development. Although most psychologists agree that the family context has a major impact on children’s social and emotional development, the mechanisms through which context impacts development are less clear [19]. Research has recognized many risk factors that affect the family context and emotional development; such as poverty, stress, low parental education, dangerous neighbourhoods [20], domestic violence [21], and number of siblings. Morris et al., [22] suggested that the family context affects the development of emotional regulation through three important ways. Firstly, children learn about emotional regulation through observational learning, modelling and social referencing. Secondly, parenting practices specifically related to emotion and emotion management, which differs by cultural variation. Thirdly, the emotional climate of the family as parenting style, the attachment relationship, family expressiveness and the marital relationship. An emotionally arousing family climate may lead to increased hypothalamic-pituitary-adrenal (HPA) axis reactivity, which, over the long-term can lead to atrophy in structures in the prefrontal cortex that play a role in emotion regulation [23]. The overall amount of emotion in the family, particularly negative emotionality, may actually induce negative emotions in early infancy and beyond [24].

### Maternal and Infant Well-being

Attachment relationships, an essential feature of socio-emotional development in infancy, are impacted by the emotional lives of caregivers and infants [25,26]. Maternal physical and mental health have a significant impact on the in utero environment and, thus, on fetal development and the health of the child later in life [27]. Maternal diabetes increases the risk of fetal anomalies, macrosomia, subsequent birth injury and hypoglycemia, all of which can negatively affect developmental outcomes in the infant. Hypertension, alone or combined with a renal or autoimmune disorder, can cause placental insufficiency and inadequate fetal growth. Infant factors include chromosomal disorders [28], prematurity [29], intra-uterine growth restriction, sensory or regulatory problems and chronic or congenital health problems [30, 31]. These risk factors will impact the type of care needed by the infant, how his caregivers respond to him, and his capacity for normal physical and mental development [32].

The objective of the present study was to assess various factors that may positively or adversely affect the socio-emotional development including, infant feeding practices, aspects of infant and maternal health and socioeconomic status in a sample of Egyptian infants.

It was previously presumed that only psychosocial risk factors possessed significant predictive capability for socioemotional development. This study assumes that socio-emotional development of infants would be equally affected by different factors among which is type of feeding in addition to other factors such as micronutrient adequacy, demographic factors (as maternal education and socioeconomic status), and host factors (as child order of birth).

Based on these assumptions, we hypothesized that children’s socioemotional development is significantly associated with breast feeding, level of maternal education and birth order.

## Subjects and Methods

### Study design

This research is a cross sectional comparative study. It was done over a period of three years starting from August 2010 till August 2013. The research focused on three groups of infants aged 6–24 months classified according to their mode of feeding during the initial six months of life into three groups: breastfed, non- breast fed (consuming other milk including fresh, tinned, and powdered milk from cows or other animals) and mixed-fed infants (both breast and artificially fed). Studied infants were enrolled in the Well–Baby Clinic of the National Research Centre or from pediatric outpatient facilities in urban Cairo.

### Sample Size Calculations

The number of infants per each group was calculated according to the national prevalence of infants who are consuming other milk including fresh, tinned, and powdered milk from cows or other animals, which were calculated according to Egypt Demographic Health Survey (EDHS).

EDHS is responsible for collecting accurate representative data about health and population at a national level. El-Zanaty and associates conduct it on behalf of the Ministry of Health and Population in Egypt at irregular intervals according to the problem required to be investigated. The national prevalence of infants who are consuming other milk was studied by EDHS, 2005 [33] in which the estimated prevalence was as follows:

1. among breast fed infants: 42.9% aged > 6—< 12 months of age, 52.4% aged 12—< 18 months of age, 49.2% aged > 18—< 24 months of age
2. among non-breast fed infants: 75.1% aged > 6—< 12 months of age, 72.9% aged 12—< 18 months of age, 65.5% aged > 18—< 24 months of age

### Subjects

The sample size was ascertained utilizing epi Info-Statcalc version 7 [34]. The aggregate number of infants who were submitted for developmental assessment was 655 infants who were chosen as follows:

- 322 predominately breast-fed.
- 240 non breast-fed (who were consuming other milk including fresh, tinned, and powdered milk from cows or other animals)
- 93 mixed fed (who were consuming artificial milk plus breast milk)

The power of this study to calculate the required sample size was set at 80% denoting probability of finding a difference when a difference really exists.

For biochemical tests, the assessment would require 185 infants aged 6 to less than 24 months with the above stated criteria (who are consuming other milk including fresh, tinned, and powdered milk from cows or other animals). This number was estimated for calculating a true difference of 15% (denoting the probability of finding a difference when no difference existed i.e. margin of error: ± 0.15). This was categorized as 105 among breast fed infants vs. 80 among non-breast fed infants [33, 34]. Accordingly, every third woman in each group was asked to provide 5 ml of her infant's blood to be tested for serum zinc, copper, iron, vitamin B12 and CBC. Those who approved to be tested were assessed in a subsample of 193 infants (133 out of 322 breast fed infants and 60 out of 333 mixed and artificially fed infants) representing 41.3% and 18% of the total sampled infants respectively.

#### Inclusion criteria

Infants were enrolled if they were over 6 months and less than 24 months of age, belonging to the middle socioeconomic status and their guardians consented to participate in the study.

#### Exclusion criteria

Infants were excluded if they demonstrated any obvious congenital anomalies, features of genetic diseases, any metabolic or neurological problems, or if their mothers had a current or past history of mental illness.

### Methods and procedures

A specific questionnaire was intended for evaluation of:

- **Socio-demographic variables**: including the order of child, maternal age, living conditions, family income, maternal and paternal education and occupation.
- **Maternal medical history**: including history of chronic diseases before or during pregnancy.
- **Perinatal history**: including gestational age, type of labor, history of delivery problems, and admission to neonatal intensive care unit.
- **Infant nutritional history**: including type of feeding during the initial six months and the time of introduction of complementary food

#### Physical examination and assessment of growth

Physical examination and assessment of growth was performed for each participant.

#### Assessment of infant development

Bayley Scales of Infant and Toddler Development (Bayley III), developed by Nancy Bayley in 2006 [35], was utilized to assess development of infants and toddlers between the age scopes of 1 month to 42 months. The Bayley scales are described as being the most widely used developmental assessment scheme [36]. Bayley-III covers five developmental domains. Cognitive, motor and language are administered with the child; interaction, social-emotional and adaptive behavior are administered with parent questionnaires. Domain subtests can be administered individually. The social–emotional scale includes items that assess the child's mastery of functional emotional skills, such as self-regulation and enthusiasm in the world: communicating needs; captivating others and setting up relationships; utilizing emotions in an interactive manner; and using emotion signals or gestures to solve problems.

Scoring of Bayley-III has been simplified from previous versions. Scoring for every item is either 1 (credit) or 0 (no credit). Scores available include raw scores, scaled scores, composite scores, percentile ranks and confidence intervals.

The measure with a series of developmental play tasks took between 45–60 minutes to administer. Raw scores of successfully completed items were converted to scaled scores and to composite scores. The scores obtained by toddlers were used to determine their performance compared with norms taken from typically developing children. The composite scores are scaled to a metric with a mean of 100 and a SD of 15, and a range from 40–160. The norm-referenced average is from 85–115.

According to the reference average social-emotional composite score (85–115), infants of this study were ordered into two groups: below average group (with socio-emotional composite score< 85) and average and above average group (with socio-emotional composite score > 85).

Validity studies comparing the Bayley–III with other tests do not show indications of Bayley–III standard score inflation. Bayley–III composite and subtest standard scores met theoretical expectations and are consistent with the results of the Wechsler preschool and primary scale of intelligence for children—Third edition (WPPSI–III), the Preschool language scale–Fourth edition (PLS–4) [37], and the Peabody Developmental Motor Scales, Second Edition (PDMS–2) [38].

The BSID-III has established reliability. Internal consistency was assessed using a split-half reliability method and shows reliability coefficients for the subscales and composite scores that range from 0.86 to 0.93 [35].

#### Biochemical assessment

Blood specimens were collected after a fasting period of 4 h for infants. Trace element-free syringes, stainless steel needles, and special trace element tubes (Becton-Dickinson) were used.

- The serum samples were separated after 10 min of centrifugation. Serum samples were diluted at a 1:6 ratio with bi-distilled water. Serum Fe, Zn and Cu concentrations were measured using an atomic absorption flame emission spectrophotometer (Varian AA 100, Australia) (213.9 nm and 324.8, respectively) [39, 40]. A standard curve was established using a commercial Zn and Cu reference (Merck KGaA Darmstadt, Germany). The coefficient of variation of the measurements was always below 5%.
- Serum vitamin B12 was assessed using the Bayer Centaur chemiluminescence method (Bayer Diagnostics, Glen¢eld, Auckland, New Zealand).
- Hematological parameters, such as hemoglobin (Hb), hematocrit (Ht), red blood cell count (RBC), and the red cell indices, mean corpuscular volume (MCV), mean corpuscular hemoglobin (MCH), mean corpuscular hemoglobin concentration (MCHC), and red cell distribution width (RDW) were performed by an automated cell counter from a tube of well-mixed EDTA-anticoagulated.
- Hemoglobin concentration < 11 g/dl, was used as cutoff point for the diagnosis of anemia [41]. It was considered that iron deficiency existed, when serum iron <45 ug/dL [42]. Other nutritional deficiencies, were assessed according to the following cutoff values; vitamin B12 <203 pg/mL, [43] zinc <65 ug/dL [44] and copper < 63.7 ug/dL. [45]

### Ethical considerations

The study complied with the International Ethical Guidelines for Biomedical Research Involving Human Subjects [46]. The Research and Ethical Committee of the National Research Centre cleared the study protocol. Informed consent was obtained from all participants involved in the study and the information obtained at the individual level was kept strictly confidential.

### Statistical methods

All test data was manipulated and analyzed by using SPSS software program version 18.0. Qualitative data were presented by number and percent. Comparisons of groups of children having average and below average socio emotional scores were done. Data was compared using Chi square and p value was calculated to determine the statistically significant difference between the two groups. Odds ratio (OR) and 95% confidence interval (CI) were also computed [OR = (*a*/*c*)/(*b*/*d*)]. [47].

The difference between the two groups was considered statistically significant when p<0.05, and considered highly statistically significant when p<0.01. Binary Logistic regression was performed to ascertain the effects of the attained significant nutritional and social variables on the likelihood that infants have a below average socio-emotional composite score. Type of feeding, time of introduction of complementary food and level of serum zinc were considered as predictors, while other significant variables as child order of birth, level of maternal education and status of maternal employment were considered as covariates. Coding of variables followed simple coding method. The dependent variable (socio-emotional composite score) was coded on a dichotomous scale into two groups: having a below average score versus having an average or above score.

All independent variables whether continuous or nominal, were transformed into categorical variables and coded as two groups (0 and 1) except type of feeding. The reference categories were: introduction of complementary food after six months, child order ≤3, high mother education, working mother, normal serum zinc level (each was coded as 1) and breast feeding (coded as 2). Other categories: introduction of complementary food before six months, child order >3, low mother education, housewife mother, subnormal serum zinc level (each was coded as 0) were anticipated as predictors of below average socioemotional score. The categorical variable (type of feeding) included 3 groups; bottle fed infants were compared to breast fed and mixed fed infants were compared also to breast fed (reference category).

## Results

Subjects were chosen and classified by sort of feeding into 3 groups: group (1) included 322 infants predominately breast-fed whose mean socio-emotional composite score was 102.81± 31.08, group (2) included 240 artificially fed infants whose mean score was 100.63± 36.72, and group (3) consisted of 93 mixed-fed infants, their mean score was 97.40 ± 33.90. No significant statistical difference (p> 0.05) could be detected between breast, formula, and mixed fed infants mean composite scores of the socio-emotional domain.

### Nutritional factors

**Fig 1:**
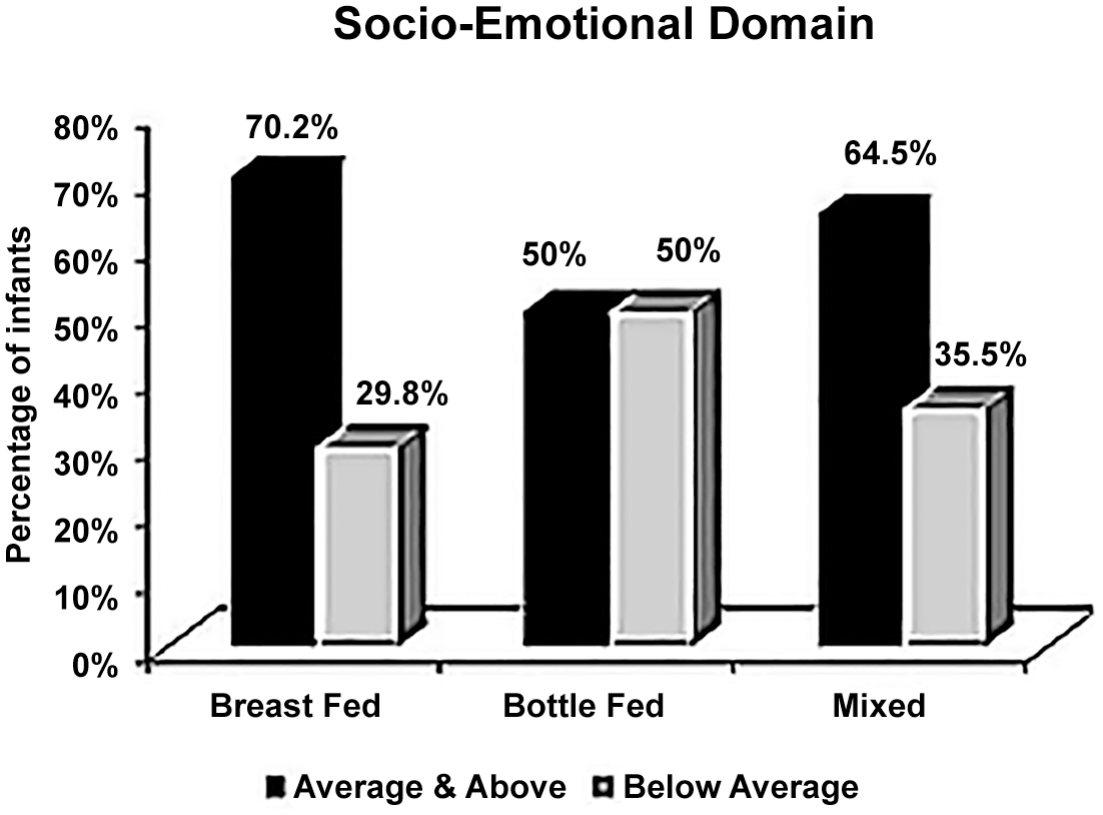
Percentage distribution of infants in each feeding group according to the reference average socio-emotional composite score in a group of 655 Egyptian infants and children aged 6–24 months old.

As shown in Fig 1, formula-fed infants were equally distributed as regard socio-emotional domain between below average class (50%) and average or above average classes (50%). This distribution was significantly different from that of the breast fed (29.8% vs. 70.2%) and mixed-fed infants (35.5% vs. 64.5%). The risk of having below average socio-emotional composite score in formula-fed infants was nearly two and half times higher than in breast fed infants (OR = 2.35, P<0.001). In the meantime, formula-fed infants had the risk of getting below average socio-emotional composite score nearly twice as much as mixed fed infants (OR = 1.82) with significant p value <0.05. Early introduction of complementary food (CF) before six months of age conveyed a significant risk of getting a below average socio-emotional composite score which was one and half times more than infants starting CF after six months of age (OR = 1.5, P <0.05). All details with respect to the impact of infant feeding practices on the socio-emotional composite score are demonstrated in Table 1.

**Table 1:** Association of infant feeding practices with socio-emotional composite scores categories in a group of 655 Egyptian infants and children aged 6–24 months old.

Parameter	n	Below Average (n = 249)	Average & above average (n = 406)	OR (95%CI)	df	X^2^	*P*-value
**Type of feeding**					2^a^	15.898 ^a^	<0.001 ^a^
*formula fed*	240	120 (50%)	120 (50%)	2.35(1.64–3.39) ^1^	1	23.685	<0.001*
Expected count (all)		91.2	148.8				
Expected count^1^		92.2	147.8				
Expected count^2^		110.3	129.7				
*Mixed fed*	93	33 (35.5%)	60 (64.5%)	1.82(1.08–3.07) ^2^	1	5.687	0.017*
Expected count (all)		35.4	57.6				
Expected count^2^		42.7	50.3				
Expected count^3^		28.9	64.1				
*Breast fed*	322	96 (29.8%)	226 (70.2%)	1.29(0.77–2.17) ^3^	1	1.083	0.298
Expected count (all)		122.4	199.6				
Expected count^1^		123.8	198.2				
Expected count^3^		100.1	221.9				
**Time of introduction of CF**							
*Before six months*	201	90(44.8%)	111(55.2%)	1.5 (1.06–2.14)	1	5.948	0.02*
Expected count		76.4	124.6				
*After six months*	454	159(35.0%)	295 (65.0%)				
Expected count		172.6	281.4				

CF: complementary food

* significant at p <0.05

^a^: Comparison between the three types of feeding; breast, bottle and mixed feeding revealed significant difference

^1^- Bottle fed vs. breastfed

^2^- Bottle fed vs. mixed fed

^3^- Mixed fed vs. breastfed

### Social factors

Social variables that influenced the likelihood of having below average socio-emotional composite score in infants are shown in Table 2. These include child order < 3, low maternal education and no maternal occupation (i.e. being a housewife). Other social factors such as maternal age and father income did not demonstrate statistically significant risk of having a below average socio-emotional composite score.

**Table 2:** Association of socioeconomic variables with socio-emotional composite scores categories in a group of 655 Egyptian infants and children aged 6–24 months old.

Parameter	n	Below average (n = 249)	Average &above (n = 406)	OR (95%CI)	df	X^2^	*P*-value
**Child's order**							
*Child's order* (>3)	443	143(32.3%)	300(67.7%)	0.48(0.34–0.68)	1	19.107	<0.001*
Expected count		168.4	274.6				
*Child's order* (≤3)	212	106(50%)	106(50%)				
Expected count		80.6	131.4				
**Maternal age**							
≤*25 years*	241	100(41.5%)	141(58.5%)	1.26(0.90–1.77)	1	1.958	0.16
Expected count		91.6	149.4				
*>25years*	414	149(36%)	265(64%)				
Expected count		157.4	256.6				
**Paternal Income** ^a^							
*Lower Middle Income*	312	126(40.4%)	186(59.6%)	1.21(0.87–1.68)	1	1.419	0.23
Expected count		118.6	193.4				
*Upper Middle- Income*	343	123(35.8%)	220(64.2%)				
Expected count		130.4	212.6				
**Maternal education** ^b^							
*Illiterate*, *read & write*	159	104(65.4%)	55 (34.6%)	2.31(1.55–3.44)	1	45.983	<0.001*
Expected count		60.4	98.6				
*High education*	496	173(34.9%)	323(65.1%)				
Expected count		188.6	307.4				
**Maternal occupation**							
*House wife*	503	203(40.4%)	300(59.6%)	1.56(1.04–2.35)	1	5.048	0.03*
Expected count		191.2	311.8				
*Working*	152	46(30.3%)	106(69.7%)				
Expected count		57.8	94.2				

^a^ Lower middle Income father: Father is unemployed, day by day worker, farmer or laborer; Upper middle Income father: Father is an employee, professional, or dealer

^b^ High Education = High School and University

* significant at p <0.05

### Maternal and infant well-being

All recorded maternal medical factors (chronic disease before or during pregnancy, malnourished or anemic mothers) or perinatal factors (gestational age, type of labor, delivery problems) did not seem to affect the probability of having a below average socio-emotional composite score in our sample. Moreover, children's medical conditions as being anemic or having subnormal serum B_12_ did not convey a risk for socio-emotional development (p>0.05). All infants had normal serum copper and normal serum iron regardless the mode of feeding. Serum zinc level appeared as a significant risk factor for socio-emotional development. The risk of having below average socio-emotional composite score in infants with subnormal serum zinc level was nearly 3 times higher than in infants with normal serum zinc level. (OR = 2.83, 95% CI (0. 97–8.64), P<0.05) (Table 3).

**Table 3:** Association of the level of infant biochemical parameters with socio-emotional composite scores categories in a group of 655 Egyptian infants and children aged 6–24 months old.

Parameter	n**	Below average (n = 249)	Average &above (n = 406)	OR (95%CI)	df	t /X^2^	P
RBCs (mean ± SD)	193	4.6±0.5	4.7±0.4			1.448	0.151
(Min.–Max.)		(3.4–5.93)	(2.8–5.6)				
HCT (mean ± SD)	193	31.6±2.4	32.0±3.8			0.86	0.391
(Min—Max.)		(28.0–36.8)	(23.1–42.0)				
MCV (fL) (mean ± SD)	193	69.0±6.8	66.7±6.9			2.170	0.031*
(Min.–Max.)		(54.0–89.3)	(47.0–85.0)				
MCH (pg/cell) (mean ± SD)	193	23.5±2.7	23.0±3.5			1.123	0.263
(Min.–Max.)		(18.0–30.0)	(15.0–43.0)				
MCHC (g/dl) (mean ± SD)	193	33.8±1.8	34.2±2.4			1.16	0.248
(Min.–Max.)		(30.0–38.3)	(29.0–51.0)				
Hb (gm/dl) (mean ± SD)	193	10.6±1.2	10.8±1.4				0.766^@^
(Min.–Max.)		(8.9–13.7)	(7.2–14.7)				
Fe (ug/dl)^4^ (mean ± SD)	193	163.0±47.4	157.4±54.2			0.701	0.484
(Min.–Max.)		(54.0–228.0)	(84.0–234.0)				
Cu (ug/dL)^4^ (mean ± SD)	193	116.7±41.2	130.0±44.6			1.969	0.056
(Min.–Max.)		(70.0–234.0)	(72.0–234.0)				
Anemia of infant ^1^							
*Yes*	89	31(34.8%)	58(65.2%)	0.94 (0.49–1.81)	1	0.061	0.85
Expected count		31.8	57.2				
*No*	104	38(36.5%)	66(63.5%)				
Expected count		37.2	66.8				
Zn (ug/dL)^2^ (mean ± SD)		83.5±31.8	101.7±47.9				0.015*
(Min.–Max.)		(36.0–162.0)	(36.0–228.0)				
*Subnormal level*	17	9(52.9%)	8(47.1%)	2.83 (0. 97–8.64)*	1	4.396	0.03*
Expected count		5.2	11.8				
*Normal level*	176	50(28.4%)	126(71.6%)				
Expected count		53.8	122.2				
vitamin B_12_ (pg/mL)^3^ (mean ± SD)		981.6±422.0	1109.4±433.3				0.028*^#^
(Min.–Max.)		(50.0–1500.0)	(50.0–1670.0)				
*Subnormal level*	9	5(55.6%)	4(44.4%)	3.35 (0.74–15.61)	1	3.392	0.06
Expected count		2.6	6.4				
*Normal level*	184	50(27.2%)	134(72.8%)				
Expected count		52.4	131.6				

^@^ independent t test

^#^ Mann-Whitney test

* significant at p <0.05

** biochemical parameters were done for a subsample of 193 children

^1^ Hemoglobin concentration < 11 g/dl was used as cutoff point for the diagnosis of anemia

^2^ cutoff point for zinc deficiency was <65 ug/dL.

^3^ cutoff point for vitamin B12 deficiency was <203 pg/Ml

^4^ iron and copper levels were within normal reference range

RBC: red blood cell count, MCV: mean corpuscular volume, MCH: mean corpuscular hemoglobin, MCHC: mean corpuscular hemoglobin concentration. HCT: Hematocrit, Hb: Hemoglobin

Table 4 includes variables that predict below average socioemotional composite score according to logistic regression analysis. It shows that subnormal level of serum zinc, **formula** feeding and introduction of complementary food before the age of six months were statistically significantly positively associated with the probability of having below average socio-emotional composite score (p<0.001, p = 0.001and p = 0.006 respectively). The table also shows that the likelihood of having below average socioemotional composite score will increase 1.15 folds if the level of serum zinc was below normal and other variables were controlled, 0.62 if complementary food was introduced before six months of age and 0.85 if the infant was **formula** fed. On the other hand, child's order of birth, mother education, mother occupation and mixed feeding were not significantly associated with the probability of having below average socio-emotional composite score (p>0.05).

**Table 4:** Logistic regression of variables predicting below average socio-emotional composite score.

	B	S.E.	Wald	df	p	Adjusted odds	95% Confidence Interval for adjusted odds	
							Lower Bound	Upper Bound
Intercept	.001	.744	.000	1	.999			
Child order	.140	.255	.300	1	.584	1.150	.697	1.897
Maternal education	.405	.264	2.351	1	.125	1.500	.893	2.518
Maternal occupation	-.345	.301	1.316	1	.251	.708	.393	1.277
Level of serum zinc: subnormal ^a^	1.154	.293	15.518	1	.000	.315	.178	.560
Introduction of complementary food: before six month ^b^	.620	.227	7.489	1	.006	.538	.345	.839
Feeding type: bottle ^c^	.848	.247	11.745	1	.001	2.335	1.438	3.793
mixed ^c^	-.048	.320	.023	1	.880	.953	.509	1.783

^a.^ Reference group: normal serum zinc

^b.^ Reference group: Introduction of complementary food after six months,

^c.^ Reference group: breast feeding.

## Discussion

Factors influencing socio-emotional development were grouped into nutritional variables, social elements and maternal and infant well-being elements.

### Nutrition

The most important nutritional factors were kind of feeding practices during the initial six months of life and the time of introduction of complementary food. As indicated by Bayley III, the socio-emotional composite score grouped infants into average and below average classes. The risk of having below average socio-emotional composite score in formula-fed infants was nearly two and half times higher than breast fed infants (OR = 2.35, P<0.001). Our outcomes are in concurrence with the findings of past studies that have analyzed the impact of breastfeeding on early indicators of children’s development [48, 49, 50, 51]. Breast milk contains a suite of nutrients, growth factors, and hormones that are important for brain development, including critical building blocks such as docosahexaenoic acid (DHA) and choline. In addition, the physical act of breastfeeding may enhance mother-infant interaction, which are important for cognitive and socioemotional development [52].

However, various research findings clarifying the impact of breast feeding on infant neurodevelopment are not consistent. In a review by Jansen et al [53], they reported that effects seen in development could not be explained by breastfeeding alone. Swain et al [54], also found that brain areas involved in parent-infant bonding and interaction are involved in other social situations such as love and attachment. Moreover, skin-to-skin contact is suggested to play a major role in maternal sensitivity [55], which has been related to better cognitive development of the child [56]. On the other hand, no significant statistical distinction could be recognized between breast fed and mixed fed infants in the likelihood of having average and above average socio-emotional composite score. This is because of the known relationship between breast milk feeding and optimal brain function even if it is not the sole source of infant nutrition, suggesting that any amount of breastfeeding during infancy is associated with points of interest on assessments of neuro-development. This is a potentially important finding as it suggests that breastfeeding is not an ‘all or none response’ and that some benefits of breastfeeding on neuro-development may be presented regardless of the possibility that the mother is not ready to provide exclusive breastfeeding for a time of 6 months [50]. Breastfeeding may enhance mental development owing to the composition of breast milk and to the act of breastfeeding, as it may foster a positive mother-infant relationship, which is important for socio-emotional development [16]. However, the pathways by which breastfeeding affects emotional development are not direct and are always intermingled [57].

The mean socio-emotional composite score was significantly higher in infants whose mothers introduced CF after 6 months of age. Likewise, the percentage of below average infants was significantly less when CF was introduced after 6 months of age. Proper complementary feeding for breastfed infants is crucial for their optimal growth and development. WHO recommended the introduction of complementary foods around the sixth month of life, instead of between the fourth and sixth month, as was previously recommended [58]. Several studies carried out in both developing and industrialized countries showed that early introduction of complementary foods increases infant morbidity and mortality, shortens the duration of breastfeeding [59], interferes with the uptake of important nutrients found in breastmilk such as iron [60], zinc [61], and reduces the efficiency of lactation in preventing new pregnancies [62].

Late introduction of complementary foods is also disadvantageous, because infant growth stops or slows down and the risk of malnutrition and micronutrient deficiency increases [63, 64].

### Social factors

Developmental outcomes are influenced to different extents by a number of risk factors such as biological, social, family and medical factors [65, 66, 67]. Children living in poverty are at risk of adverse developmental outcomes because of the aggregate impacts of introduction to hazard components as repeated infections or malnutrition. In addition, living in low financial conditions may diminish open doors for profitable learning and social communication [68]. Children’s cognitive and emotional competencies are observed to be influenced by living in impoverished socio-economic environments [69, 70].

In this study, **maternal education** was one of the influential social factors. Infants belonging to uneducated mothers had a higher risk of getting below average composite score than those belonging to highly educated mothers. This finding is in concurrence with previous studies, which reported that infants of less-educated mothers are more likely to be exposed to inadequate dietary intake, poorer sanitation [71] and receive less cognitive stimulation, than those of more-educated mothers [72]. In addition, the current results revealed that infants of housewives had a higher risk of getting below average socio-emotional composite score than infants of working mothers. This may be attributed to the sense of financial security and sense of well-being of the employed mother, which provide some sort of mother-child interactions linked to better development outcomes. This finding is similar to the results of a comprehensive meta-analysis of 69 research studies evaluating the impact of maternal employment during infancy/early childhood on child achievement and behavior problems [73]. These studies indicated that when families were at risk because of financial challenges, early maternal employment was associated with higher levels of achievement and lower levels of internalizing behaviors such as anxiety and depression. Maternal employment provided those families with financial security, lower levels of family stress and enhanced learning opportunities for children.

Moreover, **child birth order** more than the third appeared to have a positive effect on socio-emotional development; this may be because of stimulation and interaction with older siblings.

In this study, logistic regression analysis revealed the interaction between infant feeding variables and socio-economic variables in predicting infant socio-emotional outcome. Subnormal level of serum zinc, formula feeding in the first six months of life, and introduction of complementary food before the age of six months were significantly associated with the probability of having below average socio-emotional composite score. It is believed that zinc is a vital nutrient for the brain. It has an important role in neurogenesis, maturation, migration of neurons and in synapse formation [16, 74]. For each nutrient, there is a limit at which insufficiency can bring about impairment for the child. Evaluation of this level remains a vital inquiry that must be answered for each nutrient independently [16]. Although iron deficiency anemia (which affected 46% of the studied subsample) had no effect on the score, yet three studies in developing countries found altered socio-emotional outcomes in full-term infants with iron deficiency anemia when compared to infants without anemia [75, 76, 77]. These differences may be due to the variations in environmental settings, where enriched environment shields kids from negative impacts of under-nutrition. In addition, the degree of nutritional deficiency is another factor that may have an influence leading to mental deficits [16]. There was scarce evidence for the effect of other micronutrients such as vitamin B12 on the socio-emotional development of young children [78].

### Maternal and infant well-being

Various studies suggested that psychosocial risk factors influence socio-emotional development as opposed to biological risk factors of pregnancy and delivery [79, 80]. Usually prenatal and intrapartum factors and preterm labor can adversely influence the outcome for infants causing a broad range of neurodevelopmental impairments [81]. However, in this study many medical factors as maternal chronic diseases before or during pregnancy, delivery associated problems and prematurity did not affect the socio-emotional composite score. The effect of these risk factors especially prematurity could be subtle, and may be detected later on in childhood or adolescence. Symptoms suggestive of Attention Deficit Hyperactivity Disorder (ADHD), which are observed in childhood, occur two to six times more frequently in children who are born preterm [82]. On the other hand, recent advances in early neonatal care may result in improved survival of infants born preterm and ameliorate subsequent developmental and health problems [29].

It is clear that not all children exposed to risk factors will encounter adverse development. Multiple factors as family characteristics, child characteristics, socioeconomic status and sources of support outside the family will interact in a complex fashion [83].

### Limitations of the current study

This study was cross-sectional and consequently causality could not be inferred. Feeding information was based on administered questionnaires, which are subject to recall bias. Depending on interview, observation, and lack of comprehensive assessment to exclude mothers with mental health affection may disregard one of the important risk factors affecting infant socio-emotional development.

**In conclusion:** It is clear that complex interactions among multiple influences will result in great variability in infant socio-emotional development. Clear advantages of breastfeeding even if it is not exclusive are evident in this study. These advantages could be conferred to infants with mixed feeding. Maternal education stands as a strong influence on children’s development. Micronutrient sufficiency shows up as an important determinant of infant psychological well-being. Therefore, our endeavors ought to be directed towards integrated interventions addressing multiple influences of children’s development (e.g., healthy nutrition and stimulation) rather than singular interventions to prevent developmental decline in the developing world. Media should be involved in the promotion of breastfeeding, proper complementary feeding practices and increasing awareness about the importance of social and emotional development. Consideration ought to concentrate on socio-economic empowerment especially education of females. Enhancing antenatal and postnatal care can be achieved through micronutrient supplements, dietary fortification, food distribution and nutrition counseling.
